# Bridging the evidence gap: a qualitative COM-B analysis of facilitators and barriers to evaluating heat-health action plans in Germany

**DOI:** 10.1186/s12913-026-15869-1

**Published:** 2026-11-02

**Authors:** Hannah Lintener, Alice Hoeppe, Marike Andreas, Lisa Kellermann, Sophie Busalt, Peter Schäfer, Falko F. Sniehotta

**Affiliations:** 1 Division of Public Health, Social and Preventive Medicine, Center for Preventive Medicine and Digital Health (CPD), Medical Faculty Mannheim of the University of Heidelberg, Mannheim, Germany; 2 Youth Welfare Office and Health Department, City of Mannheim, Mannheim, Germany

**Keywords:** Heat-health action plan, Evaluation, Evidence-based public health

## Abstract

**Background:**

Extreme heat and heat waves are increasing in intensity, duration, and frequency. The World Health Organization recommends the development of heat-health action plans (HHAPs) for public health protection. In Germany, districts and federal states have started to implement HHAPs. However, reports on ongoing evaluations as well as evidence on the effectiveness and efficiency of planned or implemented measures are scarce. We aimed to explore the perspectives of professionals in heat and health protection on current evaluation practices, focusing on perceived facilitators of and barriers to the evaluation of HHAPs at different stages.

**Methods:**

We interviewed 23 professionals with expertise or leadership responsibility from the field of heat and health working in public administration at the district, federal state, and national level, or nongovernmental organizations (NGOs) throughout Germany. By conducting thematic analysis following a hybrid combination of deductive and inductive coding within the COM-B model, we derived and categorized facilitators of and barriers to evaluating HHAPs. Additionally, we clustered them into individual, organizational, and policy levels based on the Socioecological Model.

**Results:**

Although all interviewees perceived the evaluation of HHAPs as important, evaluation procedures were rarely systematically realized in practice. Interviewees emphasized the importance of opportunities such as political support as well as a clear mandate and assignment for evaluation. Moreover, structural conditions, like the absence of guidelines and standards for evaluation, data infrastructure, personnel, and funding, were reported to often hinder evaluation, resulting in limited experience and knowledge in evaluating complex interventions such as HHAPs. Collaboration and networks were reported as facilitative in process evaluation. While individual motivation was not reported as a reason for not evaluating HHAPs, a combination of identified barriers often contributed to feeling overwhelmed with the task of evaluation.

**Conclusions:**

Our findings suggest that evaluation is not consistently institutionalized in German heat-health action planning. Barriers to evaluating HHAPs are not primarily related to individual or district-level motivation, but rather limited by opportunity and capability shaped within political and institutional conditions. To institutionalize HHAP evaluation, greater political commitment, supportive structures, data infrastructures, and guidance for evaluation, as well as targeted funding and personnel are required.

**Supplementary Information:**

The online version contains supplementary material available at https://doi.org/10.1186/s12913-026-15869-1.

## Background

The severity, duration, and frequency of extreme heat events are increasing globally [1], with detrimental effects on human health. As of 2021, it was estimated that 37% of warmer season heat-associated deaths across 43 countries could be attributable to climate change, with impacts observed on every continent [2]. Also in Europe, heat was observed to contribute to a higher prevalence of morbidity and mortality, either directly or by exacerbating preexisting health conditions or inequalities [3]. In Germany, between 1900 and 4500 annual deaths from 2020 to 2024 were estimated to be related to heat, after a decline to around 2200 deaths in 2025, heat-attributable mortality recently surged to an estimated 16,000 deaths in 2026 [4–6].

To address the risks of rising temperatures for population health, the World Health Organization (WHO) recommends implementing heat-health action plans (HHAPs) [7, 8]. According to the WHO guidance, eight core elements were recommended for the implementation of HHAPs, including real-time surveillance of heat-related health outcomes and evaluation [7, 8]. Evaluation is a fundamental principle of public health, enabling informed decision-making on the design and implementation of programs that ensure the most effective and efficient use of resources [9]. In heat-health action planning, evaluation can focus on two key areas: processes and outcomes. Process evaluation assesses whether the HHAP was implemented as initially intended, whereas outcome evaluation examines its impact on outcomes such as mortality, morbidity, or healthcare utilization [7]. Following these recommendations, countries and districts throughout Europe are increasingly developing, planning, and implementing HHAPs to protect the population from the risks of heat [10, 11]. However, in a survey on HHAPs conducted among European Union Member States, researchers found that real-time surveillance and evaluation were among the least often implemented items from the WHO guidance on HHAPs [11]. Consequently, the number of published evaluations of HHAPs in recent years was relatively low [12], with initial approaches and evidence beginning to emerge recently [13, 14].

A 2017 guideline for implementing HHAPs in Germany identified the Public Health Service (PHS) at the federal state level as one of several potential lead agencies for coordinating HHAP development. Local districts were designated as responsible for the on-the-ground implementation of these plans, with district public health offices (PHOs), but also environmental or climate offices, as key actors [15]. This federal coordination structure reflects the multi-level organization of Germany’s PHS, encompassing agencies at national, federal state, and district levels [16]. Depending on the federal state, district PHOs are usually part of the local administration and bound by the regulations of the higher federal state-level authority [17, 18]. Despite its numerous responsibilities, particularly at the district level, it was previously estimated that the PHS only accounted for less than 1% of expenditures in the German health system [16, 18].

HHAPs have increasingly been planned, developed, and implemented throughout Germany [19, 20]. Blättner et al. [19] analyzed 375 heat measures and 19 HHAPs implemented between 2003 and 2019. They found that while recommendations for developing HHAPs were often considered, the included measures varied widely across regions. Notably, only 10 out of the 375 analyzed heat-related measures included monitoring and evaluation methods [19]. This might be due to ongoing methodological challenges such as isolating the heat-health association, the impact of individual interventions on health outcomes, or identifying a suitable control group or no-action scenario [12]. However, the limited consideration of evaluation in HHAPs might also result from underlying factors at the individual, organizational, and policy levels. Based on the same dataset, we previously identified system-level conditions as essential for enabling multi-agency and intersectoral collaboration at the district level [21], emphasizing the need to approach heat protection using multi-level frameworks [22, 23]. These insights emphasize the need to explore facilitators and barriers to the evaluation of HHAPs among practitioners involved in their planning, development, or implementation.

In this study, we extend previous findings by moving beyond documenting whether HHAP evaluations were conducted to examining the conditions perceived by stakeholders as facilitating or hindering evaluation in Germany. Specifically, we aimed to identify facilitators and barriers to evaluating HHAPs among a sample of 23 actors involved in heat-health action planning in Germany, with a particular emphasis on the PHS. Following a theory-led approach, we structured the analysis around the capability, opportunity, and motivation subcomponents of the COM-B model for behavior change [24]. Considering that HHAPs can be considered complex interventions that not only operate within a complex political environment, but also involve several administrative levels and actors, we additionally mapped themes onto levels of the Socioecological Model [25, 26] to contextualize whether the factors were described at the individual, organizational, or policy level. Finally, our qualitative findings also highlight evaluation approaches in districts and federal states that may serve as examples for other settings.

## Methods

The reporting in this paper is based on the Standards for Reporting Qualitative Research (COREQ) [27]. For this study, we applied the COM-B model to analyze interviews with 23 professionals in heat-health action planning throughout Germany to identify what facilitates or impedes the evaluation of HHAPs. The interviews were conducted with the aim of examining the role of the PHS in heat protection in Germany, however, evaluation was one of several thematic sub-modules in the interviews. Preliminary insights into the interview dataset informed a report on climate adaptation and heat protection in the PHS [28], and a recent related peer-reviewed publication specifically focused on multi-agency and intersectoral collaboration in heat-health action planning [21].

### Theoretical framework

Facilitators and barriers to evaluating HHAPs were examined within the COM-B model for behavior change. The COM-B model understands behavior as resulting from three interacting and complementary components: capability, opportunity, and motivation, each with two subcategories [24]. It has previously been applied in research aiming to understand health behavior, e.g., in the provision of health care services [29, 30] and individual health behavior [31, 32], and for developing interventions [33, 34], and can also be applied to behavior embedded in social structures, such as organizations [35, 36]. Capability includes psychological and physical abilities like knowledge and skills, whereas opportunity refers to social and physical environmental factors. Motivation involves brain processes, both automatic and reflective, that drive behavior. All of these components can also influence each other [24].

HHAPs are implemented within a complex interplay of psychological, physical, and social conditions that shape whether and how evaluation is approached and conducted. Applying the COM-B model to the evaluation of HHAPs allowed us to systematically organize these conditions according to capabilities, opportunities, and motivations, to understand the drivers or barriers underlying evaluation activities.

However, despite the frequent use of the COM-B model to examine both collective and individual behaviors, the components and sub-components do not explicitly account for a distinction between individual and collective or contextual factors. To add depth to the interpretation of COM-B-informed themes, we additionally mapped and interpreted the themes within the Socioecological Model [25], which proposes a multi-level framework encompassing not only individual, but also socio-environmental factors, and was previously extended and applied in health promotion [26], and in the context of heat protection [22, 23]. Specifically, we followed a simplified categorization previously applied in the context of HHAP implementation [22], and mapped themes into individual, organizational, or policy levels. Adapted to our study context, the individual level describes individual factors like knowledge, skills, or attitudes of individual professionals, the organizational level relates to institutional factors, like formal and informal rules and regulations within the PHS or other public agencies, and the policy level relates to local, federal state, or national level policies or politics surrounding heat-health action planning [22, 26]. Therefore, the Socioecological Model introduces conceptual depth to the identified themes and enabled us to reflect on which themes could be attributed to individual-level factors, or are rather driven at the organizational or policy level.

Combined, the COM-B and Socioecological Model enable a theory-led examination of themes underlying individual and collective behaviors within a multi-level, complex intervention and environment, and the formulation of relevant and practical policy implications.

### Research team and reflexivity

The research team brought together interdisciplinary expertise in health economics (HL), health policy (HL and MA), psychology (FFS and MA), medicine (PS), public health and health promotion (LK, AH, SB), and Landscaping & Wellbeing (AH). Several authors (PS, SB, LK, AH) have PHS experience, with PS and SB actively working in the PHS during data collection. All authors have experience in qualitative research. Senior supervision was provided by FFS (Prof. Dr., Public Health).

### Interview guide

The interview guide followed the overall objective to examine the role of the PHS in heat-health action planning, and was collaboratively developed in a workshop involving two practitioners in district-level heat-health action planning affiliated with the PHS, piloted in October 2021, and iteratively adjusted to the insights gained during data collection. The interview guide (Supplementary file 1) focused on a variety of topics related to the role of the PHS in heat-health action planning, with thematic modules covering the topics of implementation of heat protection measures, networks and collaborations, addressing heat-vulnerable groups, strengthening heat protection within the PHS, and existing resources/needs, and public communication [21].

Evaluation was not the focus of the interviews. However, experiences with and attitudes toward evaluation were inquired within the “heat protection measures” module with the question “To what extent have you already conducted evaluations of heat measures/ heat-health action plan (HHAP)?” (Topic area 2 “Implementation”, Supplementary file 1). In cases where participants were at earlier stages of planning or developing HHAPs, the question was rephrased accordingly to ask whether they were planning or considering evaluation, or whether they had observed or encountered evaluation of HHAPs in other settings. Consequently, we note that the time devoted to the topic of evaluation in the interviews varied considerably, and considering an average interview length of 41.31 min, the average time designated to evaluation was estimated to have accounted for less than one-sixth of the total interview time (i.e., approximately five to seven minutes).

### Recruitment

Germany is organized into federal states [Bundesländer] encompassing urban and rural districts [Kreisfreie Städte, Landkreise] with local administrations, e.g., PHOs or the local environmental administration. We conducted an online search for professionals in leadership positions (e.g., head of department or district office), coordinating roles (e.g., climate adaptation manager or HHAP coordinator) with professional expertise in the field of heat and health protection within the PHS, environmental administration, or nongovernmental organizations (NGOs). We also recruited participants through the networks of project partners and interviewees (*convenience sampling*).

To incorporate different perspectives, we additionally aimed to account for *purposive sampling criteria*, which included representation of professionals from all regions of Germany (North, East, West, South), rural and urban districts, and, if feasible, from the district, federal state, and national levels. We also aimed to include districts or federal states that were at different stages of HHAP implementation – including no HHAP (or intention for HHAPs), planned, developing, or implemented HHAPs.

Following ethical approval, we started recruitment in November 2023 via email or telephone and invited them to 45-minute interviews. Recruitment was stopped when saturation was judged to have been reached across the broader interview modules. To recognize data saturation, each interview was followed by discussion among HL, AH, and MA, focusing on whether the interview yielded new insights across the thematic modules. Although evaluation was a part of these discussions, saturation was not assessed specifically for the evaluation-focused analysis, and the extent to which interviews generated new insights on evaluation varied between participants.

### Data collection and transcription

Interviews were conducted by HL, AH, and MA. No other people besides the interviewer and interviewee(s) were present during the interviews. The interviews were conducted, audio-visually recorded, and automatically transcribed online via Microsoft Teams. In 2 interviews, the interviewers had a pre-existing relationship with the interviewees due to a joint project collaboration. However, these relationships were collegial and may have facilitated an open dialogue. All interviewees were informed about the scope and aim of the study before the interview. The professional motivation of interviewers for conducting the study was communicated accordingly. Lastly, the interviewees did not provide feedback on either the transcripts or the findings following the interviews. Interviewers took additional reflective notes during the interviews. Each transcript was checked for correctness within the research team. For the preparation of the manuscript, only selected anonymized and significantly shortened quotations from the transcripts with no identifiable information on the interviewee or affiliation were translated using forward-backward translation via DeepL and Google Translate, with subsequent adaptation and checking of the translation accuracy by HL and AH.

### Qualitative data analysis

Transcripts were uploaded and analyzed in MAXQDA (Version: 24.7.0) [37]. HL (first-coder), AH, and LK (joint double-coding) carried out blinded double coding of all transcripts via thematic analysis [38] following a constructivist paradigm. The analysis was conducted between March and May 2025. Coding was performed in a hybrid approach combining a deductive coding framework based on the COM-B and its sub-components with iteratively adapted inductive codes within these categories. First, we developed a coding guide based on the COM-B model to assign codes relating to facilitators and barriers of evaluation to COM-B sub-components (Supplementary file 2). We started deductive analysis using a coding tree derived from the COM-B model by independently coding a first subsample (*n* = 5) of the interviews, and had a subsequent discussion to reflect. Upon agreement on extending the tree with inductive codes within the COM-B, each coder took notes on the underlying themes within deductive categories, and we discussed the first inductively extended version of the coding tree in a group discussion. After agreement on an extended version of the coding tree, we revisited the first sample of interviews and resumed independent coding alongside regular reflections to discuss overlaps, new inductive codes, and where we situated themes within the COM-B. In the last step, we mapped the identified themes within the Socioecological Model following the same procedure of reflexive group discussions, and consensus. Coding disagreements were resolved by reaching consensus among the three coders, as documented in Supplementary file 2.

### Ethical considerations

Given the non-interventional nature of the study and its focus on professional experiences and technical matters, the risk to participants was assessed as minimal. However, due to the discussion of internal processes within interviewees’ affiliated organizations, informed consent and data protection were ensured throughout the project by obtaining written consent for participation, pseudonymization of transcripts, and secure data storage. This project was approved by the ethical committee of the Medical Faculty Mannheim at the University of Heidelberg on 31.10.2023 (Reference number: 2023 − 646) and was conducted following the Declaration of Helsinki [39].

## Results

### Sample

We contacted 33 professionals in the field of heat and health, 21 of whom agreed to participate in an interview. Of those who did not agree to participate in the study, one person cancelled the interview due to sickness, whereas the rest of the invitations were left unanswered. Two interviewees suggested being interviewed in a dyadic interview with one person from the PHS and one from an environmental administration to provide a comprehensive overview of their collaborative work in planning, developing, or implementing the HHAP. No repeat interviews were carried out.

In November and December 2023, we interviewed 23 professionals in 21 interviews, with an average length of 41.31 min. This included professionals from 15 districts, two federal states, and three national-level stakeholders. Five professionals affiliated with the district-level PHS had leadership responsibility as heads or deputy heads of the district administrative offices, whereas others were either represented by professionals working as climate protection or adaptation managers or professionals who generally coordinate health and environmental tasks in their respective offices. Of all participants at the federal state, or district level, only five were situated in northern or eastern Germany. Among the 14 professionals working with the PHS, four were affiliated with urban districts, six with rural districts, and two with PHOs responsible for both the urban and rural districts, or working at the federal state level. Of seven professionals affiliated with the environmental administration (including different variations of climate adaptation or protection units), one was operating on the national level, whereas four were working in an urban district, and two in a rural district. Additionally, two professionals represented national level NGOs that are operating in the field of heat protection. Across district or federal state level participants, only one interviewee described no intention for a HHAP, whereas six were in the planning stage, five in the development stage, and eight were already at the implementation stage.

### Status of evaluation of HHAPs in Germany

In our interviews, efforts to evaluate planned, developing, or implemented HHAPs were in their early stages. In areas where HHAPs were still in development, evaluations were rarely considered systematically. Most interviewees who reported conducting process or outcome evaluations were from urban districts or federal states that were further advanced in developing or implementing HHAPs.

Among the interviewees who had or were planning to evaluate their HHAP or individual measures, process evaluation was more common than outcome evaluation. Several participants reported process evaluations conducted alongside regular gatherings of stakeholders involved in planning and implementing the HHAP to discuss progress and challenges. Several interviewees reported a regularity of four times per year: *“This is not a one-off project with a final report*,* but meetings are held four times a year where all these people come together and everyone formulates their contribution for the current or next year and then evaluates what has happened” (I17*,* dyadic interview PHO/environmental office*,* district level).* As an example of positive experiences in conducting process evaluation and the monitoring of individual components of the HHAP, one interviewee described a structured approach using checklists for each measure as helpful: *So*,* what we do is […] look at every measure we have and see: How far have we come in this measure? There is […] a milestone plan for each measure*,* which is based on: have we*,* as the lead department*,* done everything we need to do? - Yes/No. Once we have done that*,* we move on: Has the department acting as a multiplier done everything it needs to do in this measure? Yes/No; the next stage is: Have any multipliers outside the city administration done everything they should have done? […] Then we take a look*,* and if it did not work out […]: What was the reason*,* and where were the facilitators and hindering factors?” (I20*,* PHO*,* district level).*

Outcome evaluations were rarely reported in our interviews, and when they were, they focused on monitoring excess mortality and, in few cases, heat-related hospitalization or sick leave: *“We have done this [assessment of heat mortality] intensively in [district A]. In [district B]*,* we have been looking at these figures since this summer*,* and I […] it is always important to look at what we actually mean by heat mortality” (I10*,* PHO*,* district level).*

### Facilitators and barriers to evaluation

**Fig. 1:**
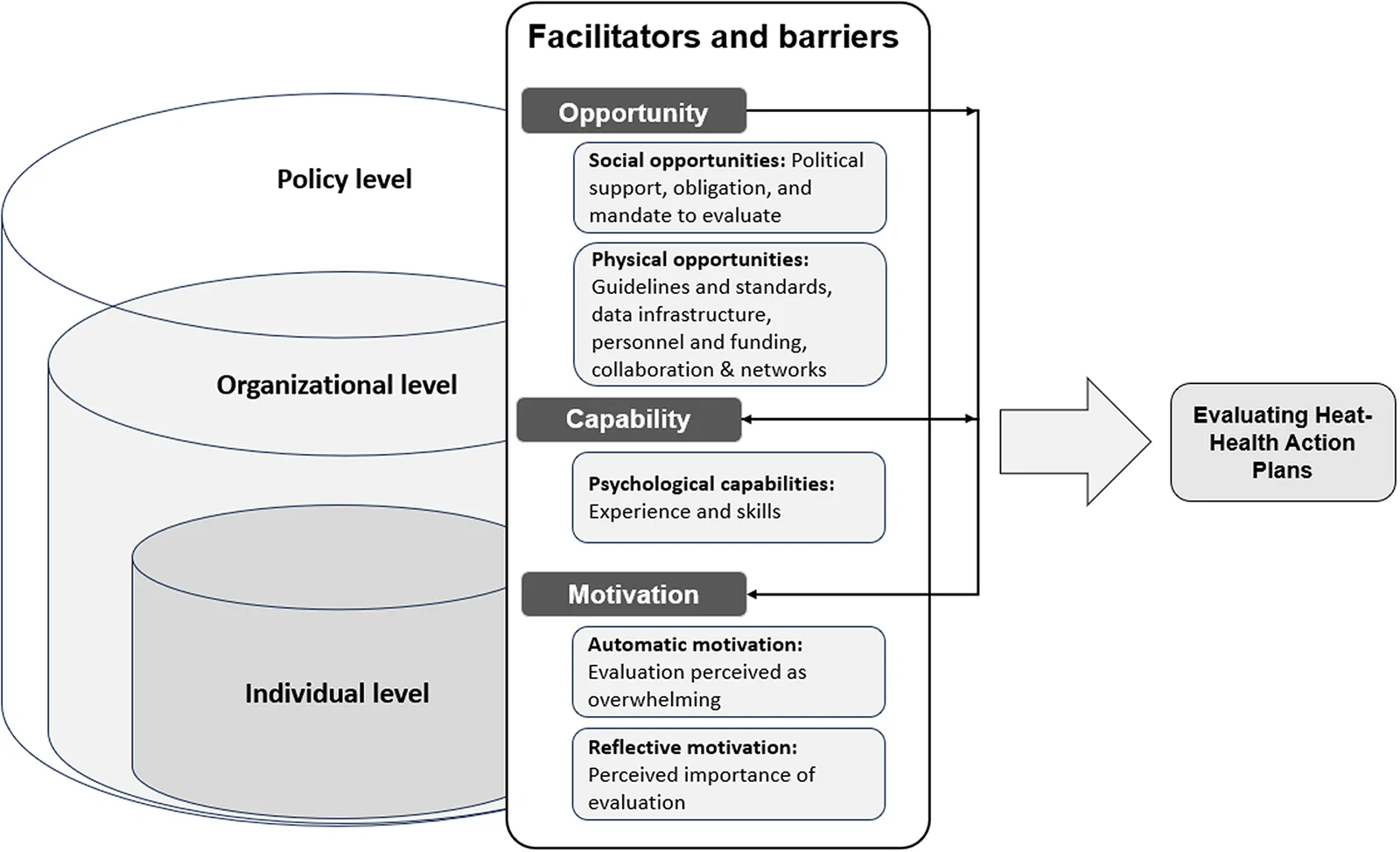
The identified themes of facilitators and barriers to evaluating heat-health action plans (HHAPs) within COM-B components and mapped within policy, organizational, and individual levels of the Socioecological Model. Please note that this figure is schematic, and several themes were mapped within more than one level of the Socioecological Model. Therefore, the levels are displayed in a nested illustration, with fluent transitions between the individual, organizational, and policy levels. The black arrows represent patterns in the findings on how the sub-components opportunity, capability, and motivation influence each other. The table underlying this figure is placed in Supplementary file 2/Table S2

Figure 1 shows the identified facilitators of and barriers to evaluation clustered within the subcomponents of psychological capabilities, social and physical opportunities, and reflective and automatic motivation.

### Capability

#### Experience and skills

Insufficient expertise or access to expertise for conducting informative evaluations among HHAP practitioners emerged as a key barrier affecting psychological capability. The interviewees reported a lack of experience and skills in the evaluation of complex interventions, i.e., in applying scientific methods and techniques for interventions such as HHAPs. They stated difficulties in dealing with scientific evidence or approaching the topic of evaluation: *“Scientific evidence is one thing*,* but it’s an area where we are not really strong because we haven’t had much to do with it yet” (I5*,* PHS*,* federal state level)* or *“I think that we always think a lot [about evaluating HHAP] and always make sure that we do a lot. But yes*,* we also come up against limits a lot” (I18*,* PHO*,* district level).* Another interviewee reported a lack of confidence in their own competency, but also no staff with the expertise to evaluate the effectiveness of recommended heat-protective measures: *“[Specific measures] are certainly in need of revision*,* but I do not have any people who can do it*,* and I’m not technically competent” (I19*,* PHS*,* federal state level)*, suggesting that capability-related barriers were described on both individual and organizational levels.

Reflecting on the organizational aspects underlying limited experience and skills for evaluation, one interviewee attributed the PHS’s emphasis on normative rather than empirical approaches to its proximity to politics as a public agency: *“[We should try] to base our work more empirically and not exclusively or predominantly on normative guidelines*,* but this is an ambitious goal because the PHS is very close to politics and simply very state-oriented” (I9*,* PHO*,* district level).* Collectively, interviewees perceived that capability issues cannot be considered as isolated from opportunities, as a lack of resources and support structures were described as influencing their perception of individual and collective evaluation capabilities.

### Opportunity

#### Political support

Interviewees considered political support essential not only for the evaluation of HHAPs but also for their development and implementation: *“It is extremely important*,* if something like this [HHAP] is to work*,* that it is scientific*,* evidence-based and informed*,* [and] that politics is of course involved”* (*I1*,* NGO*). One professional criticized that efforts or activities can be highly dependent on the political orientation of the respective ministries: *“We then decided in [year] that we wanted to evaluate this [document with instructions for heat protective measures]*,* at least as far as the technical side is concerned*,* unfortunately*,* […] this initially existing […] working group […] has fallen asleep at some point. That always has something to do with the fact that there are personnel changes in such ministries*,* that the political orientation always changes” (I19*,* PHS*,* federal state level).* Political priorities and limited institutional commitment were not only described as hindering the continuity and prioritization of evaluation activities as an essential social opportunity, but they were also described as affecting the physical opportunities, capability, and motivation across interviews.

#### Obligation and mandate to evaluate

Besides political support, multiple interviewees articulated the need for a mandate or obligation to evaluate HHAPs, calling on decision-makers to make evaluation an obligatory part of HHAPs. For instance, interviewees reported: *“Therefore*,* an evaluation*,* I think that is actually a […] problem*,* but that is what our HHAP truly lacks: an obligation to evaluate” (I20, PHO, district level).* Another participant argued: *“Just imparting theoretical knowledge is not enough. We really also need more accountability for the health authorities in general. We have [approximately] 400 independent health authorities in Germany*,* and they are [actually] not independent: they are part of the political structure and the district councils*,* and mayors […]. And if you do not want to change these structures*,* then […] perhaps it would be helpful to make very specific recommendations anyway” (I15*,* PHO*,* district level).* Considering how legislation and policies shape the responsibilities of professionals in heat-health action planning, both the need for a mandate and the obligation to evaluate HHAPs were located on policy level within the Socioecological Model. However, participants emphasized that the obligation and mandate for HHAP evaluation needs to be tied to the physical resources needed to perform this task, like personnel, and guidance.

#### Guidelines and standards

Several professionals described a lack of guidelines or standards for the evaluation of HHAPs. For instance, one interviewee noted: *“So of course*,* it is difficult: When we are doing something in a field where there is still very little good practice throughout Germany*,* then it’s also difficult to say how we can obtain the evidence” (I3*,* NGO).* This theme was also reported to influence interviewees’ perception of their own capability to conduct evaluation, as well as the perception of evaluation as overwhelming.

Several interviewees proposed ideas on how guidelines or guidance could support evaluations in local practice, describing them as a form of a “recipe” *(I10*,* PHO*,* district level)* or “checklist” *(I19*,* PHS*,* federal state level)*, possibly prepared by a committee: *“I think what we would truly need at the moment would be a guideline that is very*,* very clear in terms of professionalism*,* where it is clearly defined what is used to represent heat mortality and heat morbidity*,* […] worked out together in a certain discussion somehow*,* and this certainly requires a certain committee*,* but in the end what we truly need is something like a recipe for districts” (I10*,* PHO*,* district level).* Emphasizing the need for a standardized approach in districts, another interviewee argued: *“We are also against the health department reinventing everything*,* I do not think we need that. However*,* what we need on the ground is direct guidance for the stakeholders” (I15*,* PHO*,* district level)*, and criticized how the federalized political structure and heterogeneity of evidence-based HHAP provision in Germany contributed to inequalities in health service provision.

#### Data infrastructure

Interviewees frequently discussed data infrastructure as an opportunity for evaluation. Many interviewees described challenges in identifying or using routine data to evaluate HHAPs and monitor outcomes such as heat-related mortality. Some interviewees perceived uncertainty surrounding who would be responsible for monitoring data, or using it for evaluation. One interviewee argued that the PHS could play a key role in establishing and using data infrastructures: *“I think that is how it starts*,* to see: Where*,* where is the data? What data is collected? How can it be evaluated*,* and how can it perhaps also be partially contextualized with the knowledge of how data are collected with all its weaknesses and strengths? However*,* as health authorities*,* we are definitely the right people to do this because we collect the data ourselves” (I18*,* PHO*,* district level).* As a tool for examining local data infrastructures systematically, the inclusion of a data chapter in the HHAP was reported as helpful: *“The health department also evaluates health data*,* we have also included that [in the HHAP]*,* there is the chapter on monitoring*,* […] and everything that arises in this direction in the future*,* we would also include that*,* because it is not an acute measure*,* but it is part of the measures” (I11*,* environmental office*,* district level).*

Participants considered other physical opportunities, like collaboration with other agencies or organizations, as essential in establishing data infrastructures. Examples of data sources for monitoring and evaluation were data routinely collected or provided by health authorities, emergency services, or nursing home inspections. However, some interviewees perceived the lack of administrative structures and communication between public agencies as a challenge: *“We have many possibilities to collect information*,* also statistically*,* and to analyze it*,* we have also seen that we have collected a lot of Covid-19 data in the Covid-19 pandemic […]*,* where I thought*,* my goodness*,* this is actually statistical gold*,* and nobody wants it” (I19*,* PHS*,* federal state level)*. Another interviewee mentioned the lengthy process of setting up an infrastructure for monitoring heat mortality: *“[…] there is finally a relatively timely […] reporting on the development of heat-related mortality here in Germany […]. For this purpose*,* appropriate regulations had to be created first*,* so that the data could be transmitted from the registry offices via the PHOs or federal state statistical offices to the Robert Koch-Institute. These structures had to be created first*,* and that took some time” (I2*,* environmental administration*,* national level)*. This account illustrates how evaluation-related data infrastructure can extend beyond the local level, with political or administrative structures at national and federal state levels shaping the physical opportunities in districts.

#### Collaboration and networks

Collaboration was described as essential for HHAP evaluation. Participatory methods and structures involving local stakeholders and the population were mentioned as facilitative, particularly for process evaluation. Several interviewees stated that they had either implemented or proposed regular process evaluation meetings several times (often four times) throughout the year. For instance, an NGO representative supporting districts in implementation reported: *“I can already see movement there. That is what we’re doing in [district]. That is what we’re doing and what I think is very important*,* and there’s probably also evidence that it works if you get the key players together and simply do a review together*,* what worked*,* what did not*,* what do we need to do next” (I3*,* NGO).*

Particularly considering the perceived capability barriers reported by participants, collaboration alongside scientific institutions was perceived as facilitative for evaluation: *“And that is the same issue now for all the transformational changes*,* that we practically need researchers who go into the projects and then also develop the methods that fit and work for these complexities and then help the practitioners on the ground” (I3*,* NGO).* Despite positive experiences working alongside research partners in our interviews, those collaborations were associated with third-party funded projects. To support districts in HHAP evaluation, one interviewee also suggested joint efforts between districts alongside research partners as opposed to conducting evaluation in individual districts: *“[It is] not that everyone is trying to set up some kind of study but that you truly do it through alliances where you can also bundle resources*,* where*,* in cooperation with universities and scientific institutes*,* you simply have large numbers of cases*,* where you […] can also implement meaningful projects” (I9*,* PHO*,* district level).* However, collaboration was also described as time-intensive, and tied to personnel and funding, as well as dependent on social opportunities.

#### Personnel and funding

Interviewees identified adequate personnel capacities and the ability to allocate a sufficient amount of time as essential physical resources needed to systematically implement and evaluate HHAPs, viewing these as key elements symbolizing political support for evidence-based heat protection: *“To get such new topics [such as evaluations] off the ground*,* you need a human being*,* and that is the staff position that we simply need” (I15*,* PHO*,* district level)* or *“I actually need staff who simply have the time to deal with it” (I4*,* PHO*,* district level).* However, several interviewees emphasized a need for long-term staff positions to cope with the workload associated with heat-health action planning and climate adaptation, and enabling evaluation efforts. Several interviewees with designated staff for heat protection were acquired mostly as part of third-party funding, with a limited timeframe. While this enabled participants to progress in the evaluation of HHAPs or to collaborate with external partners, sustainable financial resources were seen as a basis for sufficient personnel and long-term commitment to the topic. Overall, personnel and funding were described as highly dependent on social opportunities and political enablement, however, they were also described as impacting motivation and capabilities on an organizational level.

### Motivation

#### Perceived importance of evaluation

All interviewees perceived evaluation as important and relevant. Interviewees expressed how it is important to gather scientific evidence of what works in practice to enable an informed HHAP: *“Next*,* we need empirical values*,* what has already become of the implemented measures*,* who has done what and how this has proven itself in practice*,* and then we need an impact evaluation” (I15*,* PHO*,* district level)*. Moreover, several interviewees argued that gathering evidence from HHAP evaluation could draw political attention to the need for investment in heat-health action planning, and may increase public acceptance of measures. Overall, our interviewees rarely described a lack of motivation as a reason for not conducting evaluation, and rather emphasized that the reasons were other factors perceived as outside of the control of individual or district administration.

#### Perception of evaluation as overwhelming

Participants frequently described the topic of HHAP evaluation as overwhelming. They reported not knowing where to start with evaluation or expressed frustration with the workload, complexity of the task, and lack of help they were receiving: *“I just believe that we are still a long way from that [guidance for the evaluation of HHAP]*,* but on the way there*,* I think it will unfortunately be the case that the districts are left on their own with their concern: Now we need to have a HHAP*,* and we do not even have time for that […]” (I10*,* PHO*,* district level).* While perceiving evaluation as overwhelming is considered an individual-level response, interviewees collectively located the conditions contributing to that feeling not only in limited experience and skills but predominantly in organizational opportunities and contextual conditions. Particularly, a lack of political support affecting funding and staff, and a lack of guidelines or standards, were described as limiting district- or federal-state-level capacities for evaluation.

## Discussion

Our qualitative COM-B analysis of interviews with 23 professionals in German heat-health action planning revealed that HHAP evaluation is still in its infancy in Germany. By interpreting facilitators and barriers within COM-B components with additional clustering within the Socioecological Model, our results suggest that the limited consideration of evaluation in HHAPs appears to be less related to individual motivation and more strongly associated with opportunity and capability shaped by political and organizational conditions.

### Interpretation

Despite collective recognition of the importance of evaluation among stakeholders, our findings emphasize that systematic evaluation remains inconsistently institutionalized in local heat protection practice in Germany, aligning with previous findings from Martinez et al. [11], Kaiser et al. [20], and Blättner et al. [19], who reported that few developed or implemented HHAPs have been evaluated. As our interviews suggest, evaluation activities are yet limited, and where they exist, they are often ad hoc or rely on individual initiative rather than embedded systems of accountability or standardized protocols.

By examining facilitators and barriers for evaluation within the COM-B and levels of the Socioecological Model, we found that social opportunities, including the absence of a clear mandate to evaluate and political support, were driven at the policy level, reflecting previous findings identifying a lack of political support or mandate as an important driver of heat-health action planning in Germany [20], multi-agency and intersectoral collaboration [21], and uncertainty surrounding the roles of public agencies, like the PHS [21, 22]. However, in our interviews, we observed that these conditions also influence local authorities’ ability to engage in evaluation efforts by limiting physical opportunities, such as underdeveloped data infrastructures, a lack of personnel and funding, and insufficient standards and guidance for evaluation. Limited experience and skills were reported to be driven by the availability of physical opportunities, further leaving individuals and districts overwhelmed with the task of evaluation, with scarce personnel and funding, and high workloads, particularly limiting capacities for evaluation.

The observed surge in heat-related mortality in the recent summer of 2026 [6] highlights the continued importance of providing evidence-based heat protection to the population in Germany. Our findings suggest the need to institutionalize evaluation and evidence use in German heat-health action planning by addressing the identified barriers through standards and practical guidance as well as long-term investments in capacities and infrastructure. Particularly in a decentralized system where federal state, and district-level decision-making dominates, these barriers to evaluating HHAPs may contribute to the observed geographic variation in the capacity to evaluate and improve heat-protection measures [19]. Coordinating the efforts of the national government, federal states, and district-level authorities is essential for leveraging synergies and establishing common standards [19, 21, 40], requiring not only administrative refinements but strategies for ensuring an efficient allocation of public resources, improving population-level health outcomes, and fostering cross-jurisdictional learning through collaboration and shared evaluation practices. The policy implications drawn from our analysis align with behavior change theory, with the Behavior Change Wheel highlighting regulation, enablement, and guidelines as interventions and policies to address barriers identified within the COM-B components [24].

Despite the fact that our findings suggest that standardized procedures and political support are needed, our study participants provided several examples and approaches of evaluation efforts in districts or federal states that could inform standardized guidance or efforts in other settings. While outcome evaluations were in the early stages in our sample, inclusion of a data chapter in the HHAP was perceived as helpful in assessing local data infrastructure. In addition to this, several interviewees in our study described positive experiences in conducting process evaluations in the form of regular reflections with all stakeholders, for instance, with checklists for each planned or implemented measure. Despite the fact that collaboration with research institutions was often facilitated by third-party funding, peer support and local networks were described as helpful to practitioners at the district level to increase social learning and create synergies amidst scarce resources.

Future studies may examine evaluation efforts in cross-regional case studies, but may also support structured approaches towards developing, advancing or piloting guidance, and tools that enable practitioners on the ground to examine the impact of heat measures. Overall, our study participants emphasized not only positive examples, but also an openness towards an increasing exchange and synergies with research institutions.

### Strengths and limitations

The strengths of our study include a sample with a broad range of interviewees, administrative agencies, and levels. The COM-B model provides a theoretical foundation for categorizing facilitators and barriers to understand the drivers of individual, but also collective behaviors. Even though the COM-B was originally developed for the observation of individual behavior change, the previous literature exemplifies the possibility of applying this model to explore collective behavior [33, 35, 36]. The Socioecological Model additionally adds depth in the form of a multi-level interpretation of themes that enable interpretation of practical implications for the policy, organizational, and individual levels. A further methodological strength is the hybrid, blinded double-coding approach, with continuous focus discussions during analysis and mapping, which increases interpretative depth.

We also note several limitations. First of all, evaluation was not the focus of the interviews, which were originally conceptualized to examine the role of the PHS in heat-health action planning. Therefore, considering the average interview duration of 41.31 min across six thematic modules, with evaluation addressed only in one module and occasional probes, the depth of discussion of evaluation was necessarily limited and varied between interviews. The findings need to be interpreted within this context, and themes may be overlapping with other topic areas, as disentangling facilitators and barriers specific to the evaluation of HHAPs from those associated with developing and implementing HHAPs is not always clear. Likewise, there may be other relevant factors to evaluation that did not come up in our interviews.

Secondly, despite the collaborative and rigorous approach to analysis, the categorization of themes to the COM-B as well as mapping into Socioecological Model levels needs to be interpreted as part of an interpretative and iterative process. Despite using a coding guide alongside reflexive discussions, and clear definitions assigned to the components and levels, the transition between sub-components of the COM-B, or levels in the Socioecological Model, can be fluid when observing complex behaviors, and overlaps are always possible. Therefore, findings need to be interpreted within this context.

Thirdly, despite aiming for a more balanced representation to include participants across urban and rural regions as well as administration levels across Germany in our sample, most participants were affiliated with the district-level PHS from the South and West of the country, and most had either planned or implemented a HHAP, reflecting the increased activity of the implementation of HHAPs in southwestern Germany reported previously [19]. However, findings need to be interpreted with caution, limiting the generalization of findings to other public agencies and across settings, like northeastern regions with differing socioeconomic and population structures.

## Conclusions

Our findings emphasize the need for a multi-level perspective that not only supports professionals and districts in developing the capability to fulfil the additional task of evaluating HHAPs, but also to create structural conditions that enable evaluation. In our study, professionals described a need for political support and an obligation to incorporate evaluation in HHAPs, but also standardized procedures, evaluation guidance, funding, and staff to fulfil the additional task, and ensure an equitable, effective, and efficient use of resources in HHAPs. Future research needs to focus on supporting standardized approaches in providing actionable, practicable, and evidence-based guidance for the evaluation of HHAPs.

## Supplementary Information

Below is the link to the electronic supplementary material.

Supplementary Material 1

Supplementary Material 2

## Data Availability

To ensure the anonymity of the participants interviewed in the study, the dataset with the interview transcripts is not publicly available.

## Abbreviations

COM-B model: Capability-Opportunity-Motivation-Behavior Model
HHAP: Heat-Health Action Plan
NGO: Nongovernmental Organization
PHO: Public Health Office
PHS: Public Health Service
WHO: World Health Organization
