# Supplementary material for: Bridging the evidence gap: a qualitative COM-B analysis of facilitators and barriers to evaluating heat-health action plans in Germany
Source: BMC Health Serv Res. 2026 Nov 2;26:1513. doi: 10.1186/s12913-026-15869-1 (PMC13732842; doi:10.1186/s12913-026-15869-1)
Supplement: Supplementary file 1 [file 12913_2026_15869_MOESM1_ESM.pdf]

Please note: The interview guideline presented in this supplementary material was previously published as supplementary material to Lintener H, Kellermann L, Hoeppe A, Andreas M, Busalt S, Schäfer P, et al. Partnerships in a heating climate: A qualitative analysis of multi-agency and intersectoral collaboration in heat-health action planning in Germany. Health Policy. 2026 Sep;171:105670. doi:10.1016/j.healthpol.2026.105670, which addresses a distinct research question based on the same dataset.

In-depth interview guide for stakeholders

Topic: Heat protection measures and the public health service (PHS)

**A. General information**		
**Question No.**	**Question**	**Option**
Survey Identification: To be filled by the researcher		
A1	Participant number	
A2	Interviewer Name	
A3	Date	[ ] [ ] / [ ] [ ] / [ ] [ ] [ ] [ ]
A4	Venue of the interview	Online
A5	Starting time	[ ] [ ]:[ ] [ ]
A6	Finishing time	[ ] [ ]:[ ] [ ]
A7	Outcome interview	1= Complete; 2= Incomplete [ ]
A8	Reason for 2 in A7 (Incomplete Interview)	

**B. Respondent Profile**		
B1	Occupation	
B2	Work Category/ Affiliation	[ ] Public Health Service<br>[ ] Environmental agency<br>[ ] University/ Research<br>[ ] NGO<br>[ ] Other professionals

Guideline

1. Introduction and aim of the project

Hello and thank you for joining us today! I'm [interviewer name] and I work as [position] at [affiliation].

In this interview, we would like to find out more about the work [with or of] the Public Health Service in the field of heat protection.

Our conversation will be recorded and transcribed in parallel by Microsoft Teams. As described in the consent form, the data will be analyzed anonymously. No information about you or your institution will be passed on to third parties. You may stop the interview at any time and do not have to answer any questions that you do not wish to answer. We have scheduled 45 minutes for the interview. Do you have any questions before we start?

**General probes**
- Wait 3 seconds]
- “How do you feel about this?”
- “Can you tell me more about it?”
- - “Why do you think that is?”

Experiences with heat protection and heat-health action planning	
**Question number**	**Formulation**
**1**	Can you start by telling us something about your work on heat protection?<br>- - How would you describe your role in heat protection?
**2 Implementation**	Which heat protection measures have you been actively involved in?<br>- At which stage of implementation are you? Planning, development, or implementation?<br>- What worked well? What were the challenges?<br>- To what extent have you already conducted evaluations of heat measures/ heat-health action plan (HHAP)? [or observed in other municipalities or districts]<br>o What worked well? What were the challenges?
**3 Networks & collaboration**	Who are you working with in regards to heat measures/ the HHAP? Who do you consider to be the most important players?<br>- When you exchange information with [named actors], what is it about?<br>- To what extent were [named actors] involved in the creation of the heat protection measures?<br>- From your “bird's eye view”: Who should the PHS work with to achieve good heat protection?<br>- What can [these actors] contribute to the work on heat protection?<br>- What obstacles have you experienced in getting in touch with stakeholders?<br>- How do you see the opportunity to introduce a Health in all Policies approach to heat protection in your municipality or district?
**4 Strengthening heat protection within the PHS (resources/ needs)**	What do you think the PHS needs to implement heat protection measures?<br>Probes (if not mentioned):<br>- What structures does the PHS need to manage heat protection tasks?<br>- What resources does the PHS need for this?<br>- What skills does the PHS need for this?

**5**<br>**Heat-vulnerable groups**	How are heat-vulnerable groups taken into account in your work/ in your HHAP?<br>- What is your impression: How (well) are these groups currently being reached?<br>- What works well?<br>- What is not working so well? Where are the difficulties?<br>- What do you think: how could these groups be reached better in the future? / What would be necessary to reach these people better?
**6**<br>**Public communication**	What methods do you observe for communicating with the population on the subject of heat?<br>- What has worked well in your experience?<br>- What are your ideas on how the local government should communicate the issues to the population?<br>- What role does the PHS play in this? What role do organizations such as NGOs or the scientific community play?<br>- Can you think of any suggestions as to how communication could be supported here, for example by the federal or state government?
**Snowball sampling**	Do you know of any other people (in your environment) involved in heat protection (especially in the PHS) who we should ask about this topic?

**C. Notes/ reflections**
Reflexions<br>C1. How well do you think the interview went?<br>C2. How much do you feel that you affected this interview?<br>C3. How much did you confess or talk about yourself in this interview?<br>C4. Please make any other notes you feel are relevant or important to add.

C5	Was the interview interrupted?	0=No, 1=Yes [ ]
C6	If yes, who interrupted?	
C7	Number of times the interview was interrupted	
