# Supplementary material for: Bridging the evidence gap: a qualitative COM-B analysis of facilitators and barriers to evaluating heat-health action plans in Germany
Source: BMC Health Serv Res. 2026 Nov 2;26:1513. doi: 10.1186/s12913-026-15869-1 (PMC13732842; doi:10.1186/s12913-026-15869-1)
Supplement: Supplementary file 2 [file 12913_2026_15869_MOESM2_ESM.pdf]

Supplementary File 2

Supplementary methods and results

Content

1. Coding guide 1
2. Documentation of coding disagreements 3
3. Final set, and categorization of themes 6

1. Coding guide

Table S1: Overview of codes: The COM-B Framework based on definitions, and quotes provided in Michie et al. 2011, slightly adapted, and interpreted within our study context.

Concept	General definition	Sub-category 1	Sub-category 2
**Capability**	**Capability**<br><br>"Capability is defined ***as the psychological and physical capacity to engage in the activity*** concerned. It includes having the necessary knowledge and skills"	**Physical capability**<br><br>**= "Skills, abilities or proficiencies acquired through practice"**<br><br>**Definition applied to study context**<br><br>Physical capability to engage in HHAP evaluation, rather understood as physical ability/ related to movement, and strength/action	**Psychological capability**<br>**= "Knowledge, memory, attention, decision processes, behavioral regulation"**<br><br>**Definition applied to study context**<br><br>Psychological capability understood as the capacity to engage in the necessary intellectual thought processes, as well as comprehension and reasoning behind HHAP evaluation and tasks associated with it
**Opportunity**	**Opportunity**<br><br>"Opportunity is defined as all the ***factors that lie outside the individual that make the behavior possible or prompt it***"	**Physical opportunity**<br>**= "Environmental context and resources"**<br><br>**Definition applied to study context**<br>Physical opportunity afforded by the environment, understood as resources in the direct	**Social opportunity**<br>**= "Social influences such as social pressure, norms, conformity, social comparisons"**<br><br>**Definition applied to study context**<br>Social opportunity afforded by the cultural milieu that determines thought processes (e.g., semantics), also

		professional environment of participants (e.g., in the Public Health Offices)	understood as institutional and political norms, and conditions in the context of HHAP evaluation (not in the immediate environment of participants, rather system-level norms, and rules)
**Motivation**	**<u>Motivation</u>**<br><br>"Motivation is defined as ***all those brain processes that energize and direct behavior,*** not just goals and conscious decision-making. It includes habitual processes, emotional responding, as well as analytical decision-making"	**<u>Automatic motivation</u> = "Emotions, reinforcement such as rewards, incentives, punishment"**<br><br>**<u>Definition</u>**<br>Automatic processes ("involving emotions and impulses that arise from associative learning and/or innate dispositions") – understood as *subconscious* thinking and thought processes underlying HHAP evaluation	**<u>Reflective motivation</u> = "Beliefs about capabilities and consequences, roles, identify, intention, goals, optimism"**<br><br>**<u>Definition</u>**<br>Reflective processes ("involving evaluations and plans") – understood as *conscious* thinking and thought processes underlying HHAP evaluation

Please note: inductive themes were categorized as COM-B sub-components on the basis of Table S1, however, an overview of discussions underlying the categorization, distinction, and naming of inductive themes is presented in "2 Documentation of coding disagreements", and was conducted on the basis of reflective notes, and discussion. The final categorization is presented in Table S2.

References

Michie, S., Van Stralen, M. M., & West, R. (2011). The behaviour change wheel: A new method for characterising and designing behaviour change interventions. *Implementation Science*, *6*(1), 42. https://doi.org/10.1186/1748-5908-6-42

2. Documentation of coding disagreements

The overarching coding disagreements, interpretations, and consensus-based agreements throughout the analysis are presented here, structured according to every step of the hybrid analysis approach.

(1) Deductive coding based on initial COM-B coding tree

In the first step of analysis, we tested the initial COM-B coding tree by independently coding facilitators and barriers of evaluation based on the same subsample of interviews. Following coding, subsequent discussions presented several coding disagreements with a need for conceptual refinement. Codes where coders were unsure were coded in a separate "to be discussed" category to enable a rich discussion. In particular, this included conceptual refinement in differentiating sub-components of the COM-B. The coders generally agreed upon the overarching differentiation between capability, opportunity, and motivation, while remaining nuances or codes that could apply to several categories were discussed in the group. However, particularly differentiating between sub-components raised several overarching discussions:

- Differentiating reflective and automatic motivation. This was resolved by revisiting the literature and reflecting upon whether the underlying motivational factors were based on conscious reflection upon the topic, or rather subconscious to the individual. We also discussed whether physical capability was applicable in our study context, and agreed upon not having identified this theme in the data yet.
- Differentiating social and physical opportunities was raising challenges as the distinction between political norms, rules, and laws raised questions. In the discussion, we resolved issues by differentiating the larger contextual, social structures, and norms (e.g., lacking support at the policy level) as a social opportunity, whereas physical resources (e.g., staff), were considered in more immediate proximity to participants, resources they would encounter or need in evaluating HHAPs in their professional role or everyday lives, e.g., guidance materials as a physical resource.

(2) Formulating inductive categories within the COM-B

In the second step, each coder reflected upon inductive categories within the COM-B that were already arising from the deductive coding, and took notes on underlying themes within the COM-B. Coders discussed the formulation of themes and agreed on a common structure, which we then resumed coding under iterative adjustment in the regular reflections. Coding disagreements in this iterative process included the following:

- Following the first discussions, we agreed that themes would be mutually exclusive, and no theme will be categorized within two COM-B components. However, we acknowledged conceptual overlaps in the interpretation, particularly in how factors interact with each other.
- Disagreements and discussion about whether codes were specific to evaluation, or rather in the context of implementation. This was discussed on the basis of several examples, which led us to agree on coding sections we considered to discuss evaluation thematically or broadly, and excluding others that we revisited upon reading them in the context of the full section in the transcripts. Slight overlaps were considered part of the iterative, interpretative process.
- Categorization *of "personnel and funding"* as social or physical opportunity. Considering that these constitute physical resources that only indirectly arise from the social context, and norms (represented by political support and obligation or mandate to evaluate), we agreed upon categorizing this theme as a physical opportunity.
- Reflection upon whether "*Experience*" would constitute a capability, or rather an opportunity. After revisiting specific sections that we assigned with this code, coders agreed upon a joint *"Experience and skills"* theme in psychological capability. We agreed that, while it can be influenced by opportunities, we consider it a capability factor.
- Dissecting reflective, and automatic motivation required discussions and reflections – however, after revisiting code snippets, and reflecting together, we agreed that *"feeling overwhelmed"* would often present as a rather subconscious feeling, whereas *"perceiving evaluation as important"* is usually associated with reflective and conscious reasoning. Consequently, the latter is categorized as reflective, whereas *"feeling overwhelmed"* was considered automatic.

In a joint extended workshop, the coders met and agreed upon the final set of themes and refined their formulation, differentiation, and whether they were actually situated in the right sub-component in the COM-B based on Table S1.

(3) Mapping facilitators and barriers according to the Socioecological Model

The major discussion points in mapping the identified themes according to individual, organizational, and policy levels of the Socioecological Model were the following:

- In mapping the identified themes according to the Socioecological Model, we realized that we need to differentiate between where the facilitators and barriers occur or are described, and where they need to be addressed. We resolved this discussion by limiting the mapping of themes according to the Socioecological level at which they

occur, as opposed to the level of potential prospective intervention. However, in many cases, there is substantial overlap between where themes occur and where intervention could be intended or was discussed in the interviews. This interpretative nuance was discussed prior to formulating policy implications as presented in the discussion section.

- We also discussed whether it is applicable to assign themes to multiple levels of the Socioecological Model, and revisited the codes assigned to these categories for examples, and contextualization. Upon discussion, we agreed that several themes were described to occur in several levels (e.g., *feeling of overwhelm* in individual professionals, but also described as a collective factor in departments or working groups that work on HHAPs).
- *Guidelines and standards* were discussed to be a mix of the policy, and organizational level, depending on whether this is presented in local efforts, on federal state level, or following a national, united initiative. We finally agreed to categorize this as policy level, as the sentiment described by most participant related to the need for guidance on the national level to decrease both the burden of and the heterogeneity between federal states, and districts.
- *Collaboration and networks:* We discussed whether this physical opportunity would constitute the organizational level, as most interviewee reports related to district level, or in some cases, federal state level collaboration. However, given that the structural, politically determined conditions surrounding collaboration were discussed frequently, we considered this both political, and organizational.

3. Final set, and categorization of themes

Table S2: Facilitators and barriers of evaluation clustered according to the COM-B. Please note, that Socioecological levels are considered to transition fluently, therefore, several levels can be applicable to each theme, whereas the COM-B themes are categorized mutually exclusive.

COM-B	Subcategories	Subthemes	Socioecological domain
**Capability**	***Psychological***	Experience and skills	Individual & organizational
**Opportunity**	***Social***	Political support	Policy level
		Obligation and mandate to evaluate	Policy level
	Physical	Guidelines and standards	Policy level
		Data infrastructure	Organizational & policy level
		Personnel and funding	Organizational & policy level
		Collaboration and networks	Organizational & policy level
**Motivation**	***Reflective***	Perceived importance of evaluation	Individual & organizational level
	Automatic	Evaluation perceived as overwhelming	Individual & organizational level
